# Dual-Function Exocytosis Regulator Has Yet Another Job

**DOI:** 10.1371/journal.pbio.1002268

**Published:** 2015-10-05

**Authors:** Richard Robinson

**Affiliations:** Freelance Science Writer, Sherborn, Massachusetts, United States of America

## Abstract

A new study shows that synaptotagmins, known to regulate the fusion of neurotransmitter vesicles with synaptic membranes, are also needed for the prior tethering and priming of vesicles. Read the Research Article.

Neurotransmitter release at the axon terminal depends on a highly orchestrated set of cell processes that collect and maintain a readily releasable pool (RRP) of transmitter-containing vesicles just below the surface of the terminal’s plasma membrane and trigger fusion of the vesicles with the membrane in response to an action potential, all the while preventing stray vesicles from fusing on their own in the absence of the appropriate cue. While the players in this orchestra, and even the part each performs, are mostly known, there are still some surprises to be found. In this issue of *PLOS Biology*, Taulant Bacaj, Dick Wu, Thomas Südhof, and colleagues show that a principal player in the last two of these processes also plays a vital supporting role in the first.

The synaptotagmins are best known as the calcium-sensitive triggers for exocytosis; they also actively prevent vesicle fusion when calcium is absent. But they have not been suspected of playing a role in the earlier steps, in which vesicles are collected at the plasma membrane (tethering) and then docked with it (priming), in preparation for release. The belief that there was no role for the synaptotagmins in tethering and priming came from deletion experiments, in which loss of any individual synaptotagmin (there are a healthy handful involved in different aspects of exocytosis and in different cell types) did not diminish the size or rate of formation of the readily releasable vesicle pool. On the other hand, several lines of evidence seemed to hint they may play a role in priming nonetheless, and so the authors set out to explore the effect of deleting multiple synaptotagmins at the same time.

They chose to work in forebrain neurons, which rely primarily on synaptotagmin-1 (Syt1), for fast calcium-triggered exocytosis, and synaptotagmin-7 (Syt7), for slower calcium-triggered exocytosis. They found that deletion of both proteins caused a 60% reduction in the RRP (Fig 1). This immediately suggested that Syt1 and Syt7 act redundantly in this process (they are not redundant in their exocytosis function), and that the ability of each to compensate for the loss of the other explained the results of the earlier single-deletion experiments.

**Fig 1:**
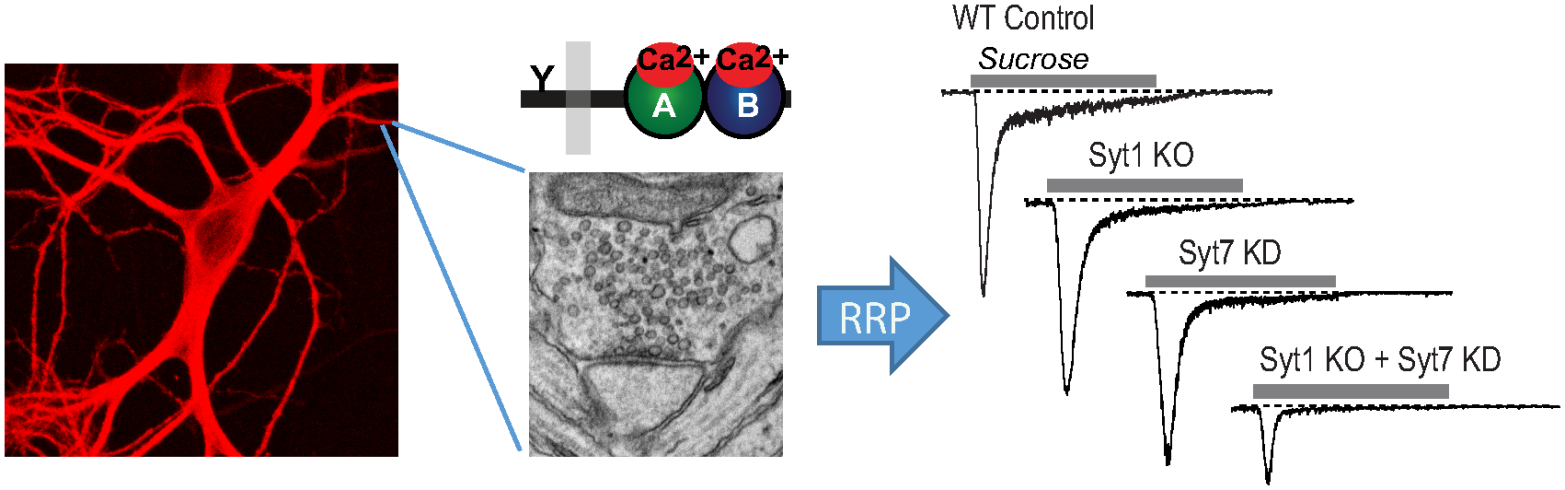
Simultaneous ablation of Syt1 and Syt7 reduces the size of the RRP of synaptic vesicles. Image credit: Dick Wu, Xinran Liu, and Thomas Südhof.

Transduction of the calcium signal by the synaptotagmins relies on cytoplasmic domains called C2A and C2B, with Syt1 relying mainly on C2B and Syt7 mainly C2A. The authors found that in Syt1, mutation to inactivate calcium binding in either domain did not interfere with its priming function. In contrast, in Syt7, inactivation of calcium binding in C2A, though not C2B, did interfere with priming. As the authors noted, this is surprising, since “although the functions of Syt1 and Syt7 in priming are redundant, their mechanisms appear to differ.”

What role do the synaptotagmins play in priming? A group of proteins called the SNARE complex links the vesicle to the membrane, and the authors showed that as long as the cytoplasmic domains were intact, both Syt1 and Syt7 coprecipitated with SNARE proteins, indicating they probably link directly with them in vivo. Syt1 increased SNARE complex assembly as long as complexin, an essential synaptotagmin cofactor, was present, an effect that did not rely on calcium. While loss of both Syt1 and Syt7 decreased the size of the RRP, it did not slow the rate of refilling of that (much smaller) pool, suggesting that the synaptotagmins likely stabilize the primed state of synaptic vesicles through their interaction with SNARE complexes, rather than facilitating formation of the complex itself.

The precise mechanistic interactions of the synaptotagmins with SNARE proteins will have to await further studies. It will be interesting to see exactly how Syt1 and Syt7 compare in the details of those interactions, given that they appear to stabilize the pool through slightly different mechanisms. It seems likely that a better understanding of these processes may also shed light on the how the synaptotagmins cooperate with SNARE proteins and complexin to carry out their two other functions, preventing vesicle fusion when calcium is absent and promoting it when it is present.

## Abbreviations

RRP: readily releasable pool
Syt1: synaptotagmin-1
Syt7: synaptotagmin-7

## References

[1] Bacaj T, Wu D, Burré J, Malenka RC, Liu X. Synaptotagmin-1 and -7 Are Redundantly Essential for Maintaining the Capacity of the Readily-Releasable Pool of Synaptic Vesicles. PLoS Biol. 2015;13(10): e1002267. 26437117 10.1371/journal.pbio.1002267 PMC4593569

